# Correlation and risk factors of peripheral and cervicocephalic arterial atherosclerosis in patients with ischemic cerebrovascular disease

**DOI:** 10.1038/s41598-024-62092-1

**Published:** 2024-05-23

**Authors:** Lu-guang Li, Xin Ma, Xiaoxi Zhao, Xiangying Du, Chen Ling

**Affiliations:** 1 Department of Neurology, Xuanwu Hospital, Capital Medical University, No. 45 Changchun Street, Xicheng District, Beijing, 100053 China; 2 National Clinical Research Center for Geriatric Disorders, Beijing, China; 3 Clinical Center for Cardio-Cerebrovascular Disease of Capital Medical University, Beijing, China; 4 Department of Radiology, Xuanwu Hospital, Capital Medical University, Beijing, China; 5 Department of Vascular Ultrasound, Xuanwu Hospital, Capital Medical University, Beijing, China

**Keywords:** Cerebrovascular disorders, Risk factors, Cerebrovascular disorders, Neurovascular disorders

## Abstract

Patients with ischemic cerebrovascular disease (ICVD) frequently develop concomitant peripheral artery disease (PAD) or renal artery stenosis (RAS), and multiterritorial atherosclerotic patients usually have a worse prognosis. We aimed to evaluate the status of peripheral atherosclerosis (AS) and cervicocephalic AS (CAS) in ICVD patients with AS, their correlation, and related risk factors contributing to coexisting cervicocephalic-peripheral AS (CPAS). Based on the severity and extent of AS evaluated by computed tomography angiography and ultrasound, the degree of AS was triple categorized to assess the correlation between CAS and PAD/RAS. CAS and PAD/RAS were defined as the most severe stenosis being ≥ 50% luminal diameter in cervicocephalic or lower limb arteries, and a peak systolic velocity at the turbulent site being ≥ 180 cm/s in the renal artery. Among 403 patients with symptom onset within 30 days, CAS, PAD, and RAS occurrence rates were 68.7%, 25.3%, and 9.9%, respectively. PAD was independently associated with the degree of extracranial and intracranial CAS (*p* = 0.042, OR = 1.428, 95% CI 1.014–2.012; *p* = 0.002, OR = 1.680, 95% CI 1.206–2.339), while RAS was independently associated with the degree of extracranial CAS (*p* = 0.001, OR = 2.880, 95% CI 1.556–5.329). Independent CPAS risk factors included an ischemic stroke history (*p* = 0.033), increased age (*p* < 0.01), as well as elevated fibrinogen (*p* = 0.021) and D-dimer levels (*p* = 0.019). In conclusion, the occurrence rates of RAS and PAD in ICVD patients with AS is relatively high, and with the severity of RAS or PAD increase, the severity of CAS also increase. Strengthening the evaluation of peripheral AS and controlling elevated fibrinogen might be crucial for preventing and delaying the progression of multiterritorial AS in ICVD patients with AS, thereby improving risk stratification and promoting more effective prevention and treatment strategies.

## Introduction

Ischemic cerebrovascular disease (ICVD) is a major cause of morbidity and mortality worldwide^1^, often attributed to cerebral arterial atherosclerosis (AS)^2^. AS is a systemic disease that often affects multiple vascular beds, primarily including cervicocephalic, coronary, lower limb, and renal arteries^3^. Research has established a connection between coronary AS and cervicocephalic atherosclerosis (CAS) in ICVD patients^4,5^. However, studies about the relationship between peripheral AS and CAS remains limited. Among peripheral atherosclerotic diseases, peripheral artery disease (PAD) and renal artery stenosis (RAS) have higher occurrence rates and more unfavorable outcomes^6–8^. Previous studies have provided initial evidence of the correlation between PAD and CAS in ICVD patients^9^, but the association between RAS and ICVD remains controversial^9–13^. Moreover, comprehensive investigations into the relationship of the severity between peripheral AS and CAS have been lacking, while such research is vital for improving our understanding of systemic AS. Furthermore, although AS in various arterial territories shares several common risk factors^12^, the factors for coexisting cervicocephalic-peripheral atherosclerosis (CPAS) in ICVD patients have not been well understood, poseing challenges for early prevention and intervention. In this study, we used peripheral arterial ultrasonography and cervicocephalic computed tomography angiography (CTA) to assess AS severity and extent among different vascular beds, and CAS, PAD, and RAS in this study were all defined as significant AS detected in the respective arterial region. Our aim was to explore the correlation between CAS and PAD/RAS and to identify the influencing factors of CPAS in ICVD patients, so as to enhance our comprehension of the interaction between AS in different vascular beds, ultimately to promote more effective preventive strategies and targeted interventions for such patients.

## Methods

### Study population and data collection

This study analyzed the data from a cohort study. We consecutively enrolled ICVD patients admitted to Xuanwu Hospital between June 2020 and July 2022. All participants provided informed consent. Inclusion criteria required patients aged between 18 and 80, with atherosclerotic lesions (plaque, stenosis or occlusion) detected in any arterial territory, and a confirmed diagnosis of ischemic stroke (IS) or transient ischemic attack (TIA) onset within 30 days based on cranial computed tomography (CT) or magnetic resonance imaging (MRI) scans. Exclusion criteria included non-atherosclerotic arterial stenosis (arterial dissection, arteritis, etc.), cardioembolism stroke, stroke of other determined etiology, and stroke of undetermined etiology in TOAST classification^14^, patients who had undergone cervicocephalic arterial surgery, and those unable to complete the required examinations within 7 days of admission.

We gathered comprehensive baseline data and medical histories from patients, including age, gender, overweight status (body mass index (BMI) ≥ 25 kg/m^2^), and history of hypertension, diabetes, smoking, alcohol consumption, IS, and coronary artery disease. Blood tests were performed on the second day after admission, including glucose, glycated hemoglobin, total cholesterol, triglycerides, low-density lipoprotein, high-density lipoprotein, homocysteine, C-reactive protein, apolipoprotein A1, apolipoprotein B, fibrinogen, D-dimer, neutrophil count, creatine kinase, and creatinine. Patient were assessed using the National Institute of Health Stroke Scale (NIHSS). All IS patients were classified according to the TOAST classification for stroke etiology^14^.

### Assessment of atherosclerosis characteristics

Within 7 days of admission, all patients underwent cervicocephalic CTA and lower limb and renal arterial Doppler ultrasound examinations to screen for significant AS. Cervicocephalic arteries were categorized into extracranial arteries (including bilateral subclavian arteries, common carotid arteries, extracranial cervical arteries, and extracranial vertebral arteries) and intracranial arteries (including bilateral intracranial cervical arteries, intracranial vertebral arteries, anterior cerebral arteries, middle cerebral arteries, posterior cerebral arteries, and basilar arteries)^15^. Dual-source 192-slice spiral CT (Light Speed, General Electric Company) was employed for cervicocephalic CTA scans, and images were reviewed by two radiologists who were blinded to clinical data. Lower limb arteries (including bilateral common femoral arteries, deep femoral arteries, superficial femoral arteries, popliteal arteries, anterior tibial arteries, posterior tibial arteries, and peroneal arteries) and renal arteries were evaluated through dual ultrasound (Philips IU22 and HDI5000) examinations conducted by two technicians. CAS, PAD, and RAS were diagnosed when significant stenosis was detected in the corresponding areas, which was defined as the most severe stenosis at any segment being ≥ 50% luminal diameter or luminal occlusion in cervicocephalic or lower limb arteries, and a peak systolic velocity (PSV) at the turbulent site being ≥ 180 cm/s (corresponding to luminal stenosis diameter ≥ 60%)^16^ or occlusion in renal artery.

The degree of AS in the four arterial territories (extracranial, intracranial, lower limb and renal arterial territories) was separatively classified as mild (without a significant atherosclerotic lesion throughout the assessed arterial territory, moderate (with a significant atherosclerotic lesion in a single arterial segment in the assessed arterial territory), and severe (with a significant atherosclerotic lesion in multiple arterial segments in the assessed arterial territory). Patients were categorized into groups based on the presence or absence of peripheral AS and CPAS, resulting in groups with peripheral AS or without peripheral AS, and CPAS group or non-CPAS (NCPAS) group.

### Statistical analysis

Statistical analysis was conducted using SPSS (v19.0; IBM). Normally distributed continuous variables were presented as mean ± standard deviation (M ± SD), while categorical variables were expressed as counts (%). Non-normally distributed continuous variables and ordinal variables were represented as medians and interquartile ranges [M (Q25, Q75)]. Group comparisons for univariate analysis were performed using t-tests, chi-squared tests, or Mann–Whitney U tests as appropriate. A multivariable logistic regression model was applied to fit variables that showed significant differences in univariate analysis (*p* < 0.1). Adjusted odds ratios (ORs) and their corresponding 95% confidence intervals (CIs) were estimated. Statistical significance was defined as *p* < 0.05.

### Ethical approval

This study was performed in line with the principles of the Declaration of Helsinki. Approval was granted by the Ethics Committee of Xuanwu hospital. (Ethics approval number: Clinical study verify [2022] No.008-revise1).

### Consent to participate

Informed consent was obtained from all individual participants included in the study.

## Results

**Figure 1:**
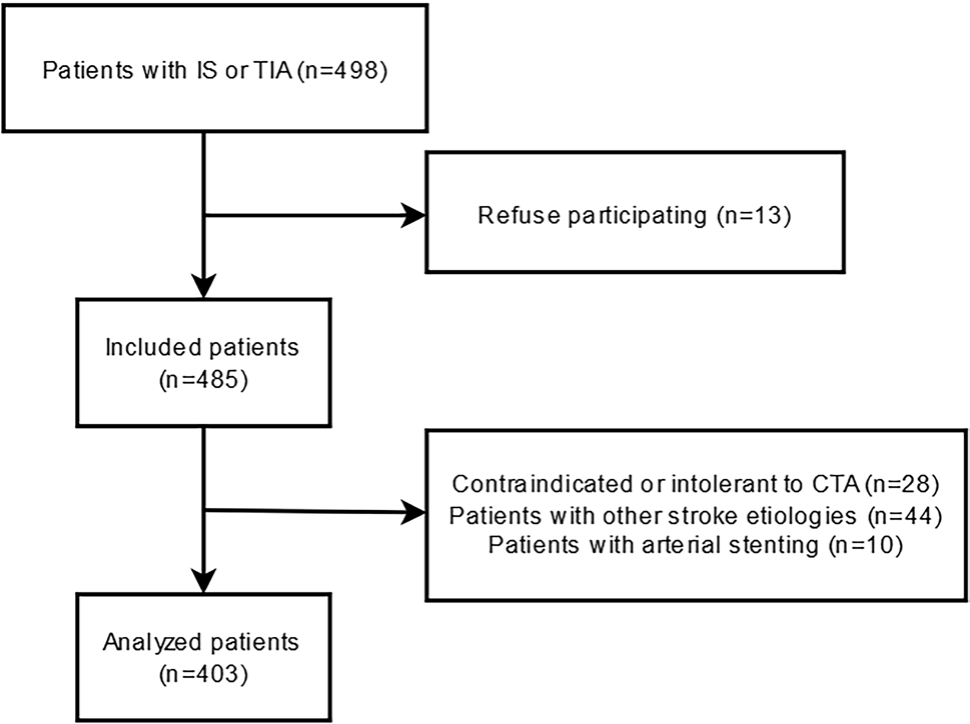
Flowchart of patient’s enrollment. A total of 498 ICVD patients met the inclusion criteria. After excluding 44 patients with other stroke etiologies, 10 patients with arterial stenting, 13 patients who refused to participate, and 28 patients contraindicated or intolerant to CTA, finally, a total of 403 patients were included in the study.

A total of 403 patients were included in the final analysis (Fig. 1), consisting of 350 (86.8%) IS patients and 53 (13.2%) TIA patients. The average age was 61.0 ± 11.4 years, with a predominance of males (69.7%) (see Supplementary Fig. S1 online).

Table 1 presents the distribution of AS in different vascular beds among the patients, the ratios of intracranial atherosclerosis (ICAS), extracranial atherosclerosis (ECAS), PAD, and RAS were 60.8%, 33.7%, 25.3%, and 9.9%, respectively. In 27.8% (n = 112) of patients, no arterial stenosis was detected in any of the examined vascular beds. CAS and PAD patients had a higher proportion of multi-segment involvement (severe group), while RAS patients predominantly exhibited unilateral involvement (moderate group).

**Table 1:** AS status of different vascular beds in ICVD patients.

	Mild	Moderate	Severe
ECAS	267(66.3%)	79(19.6%)	57(14.1%)
ICAS	158(39.2%)	93(23.1%)	152(37.7%)
PAD	301(74.7%)	44(10.9%)	58(14.4%)
RAS	363(90.1%)	33(8.2%)	7(1.7%)

ICVD, ischemic cerebrovascular disease; ECAS, extracranial atherosclerosis; ICAS, intracranial atherosclerosis; PAD, peripheral artery disease; RAS, renal artery stenosis.

### Relationship of PAD, RAS, and CAS

The coexistence of PAD and RAS increased the likelihood of developing CAS in ICVD patients by 30.1% (91.2% vs. 61.1%, *p* < 0.01) and 15.3% (82.5% vs. 67.2%, *p* = 0.048) compared to patients without PAD/RAS, respectively. Patients with CAS also had a higher prevalence of concomitant PAD (33.5%) and RAS (11.9%) compared to those without CAS. Furthermore, patients with PAD or RAS had a higher rate of AS involvement in both intracranial and extracranial vascular beds (41.5% vs. 18.9%, *p* < 0.01). This correlation appears to be unrelated to the location of PAD or CAS (extracranial or intracranial, anterior circulation or posterior circulation). We further divided the lower limb arteries into proximal (above the popliteal artery, PADP) and distal (below the tibial artery, PADD) parts. Results showed that patients with concurrent PADP (ICAS: 85.3% vs. 73.5%, *p* = 0.002; ECAS: 61.8% vs. 31.2%, *p* < 0.01) or PADD (ICAS: 80.9% vs. 55.1%, *p* < 0.01; ECAS: 51.7% vs. 28.7%, *p* < 0.01) had higher rates of ICAS and ECAS occurrence. Additionally, ICVD patients with concurrent PADP or PADD had higher rates of CAS in the anterior circulation (PADP: 76.5% vs. 52.6%, *p* = 0.007; PADD: 79.8% vs. 47.5%, *p* < 0.01) and posterior circulation (PADP: 91.2% vs. 45.8%, *p* < 0.01; PADD: 79.8% vs. 41.1%, *p* < 0.01).

After adjusting for age, gender, and clinical variables associated with the elevation of ICAS or ECAS degree in univariate analysis (see Supplementary Fig. S2 online), multivariable logistic regression showed that the degree of PAD was independently positively associated with the degree of ECAS (*p* = 0.042, OR = 1.428, 95% CI 1.014–2.012) and ICAS (*p* = 0.002, OR = 1.680, 95% CI 1.206–2.339). On the other hand, the degree of RAS demonstrated an independent positive association with the degree of ECAS (*p* = 0.001, OR = 2.880, 95% CI 1.556–5.329), while no significant relationship with the degree of ICAS was observed (*p* = 0.146).

### Risk factors for CPAS in ICVD patients

After adjusting for risk factors associated with peripheral AS and CPAS in univariate analysis (see Supplementary Fig. S3 and Supplementary Fig. S4 online), multivariable logistic regression revealed that age (*p* < 0.01, OR = 1.066, 95% CI 1.038–1.096), hypertension (*p* = 0.048, OR = 1.836, 95% CI 1.005–3.356), IS (*p* = 0.033, OR = 1.876, 95% CI 1.054–3.340), fibrinogen (*p* = 0.021, OR = 1.446, 95% CI 1.058–1.978) and D-dimer (*p* = 0.019, OR = 1.076, 95% CI 1.012–1.145) are independently associated with peripheral arterial AS. Furthermore, age (*p* < 0.01, OR = 1.068, 95% CI 1.038–1.099), diabetes (*p* = 0.019, OR = 2.122, 95% CI 1.130–3.984), IS (*p* = 0.008, OR = 2.240, 95% CI 1.236–4.059), fibrinogen (*p* = 0.012, OR = 1.512, 95% CI 1.094–2.089) and D-dimer (*p* = 0.016, OR = 1.081, 95% CI 1.014–1.152) are independently associated with CPAS (Table 2).

**Table 2:** Independent influencing factors of peripheral AS and CPAS in ICVD patients.

	Characteristics	*p*	OR(95% CI)
Peripheral AS	Age History of HTN History of IS Fibrinogen D-dimer	< 0.01 0.048 0.033 0.021 0.019	1.066(1.038–1.096) 1.836(1.005–3.356) 1.876(1.054–3.340) 1.446(1.058–1.978) 1.076(1.012–1.145)
CPAS	Age History of DM History of IS Fibrinogen D-dimer	< 0.01 0.019 0.008 0.012 0.016	1.068(1.038–1.099) 2.122(1.130–3.984) 2.240(1.236–4.059) 1.512(1.094–2.089) 1.081(1.014–1.152)

AS, atherosclerosis; CPAS, coexisting cervicocephalic-peripheral atherosclerosis; ICVD, ischemic cerebrovascular disease; HTN, hypertension; IS, ischemic stroke; DM, diabetes mellitus.

## Discussion

Despite the higher prevalence of PAD and RAS in ICVD patients compared to the general population^3,11,17^, clinical evaluation of these comorbidities remain inadequate. The peripheral arteries of ICVD patients are often not given as much attention and evaluation as the cervicocephalic arteries, leading to a possibly inaccuracute data regarding the prevalence of peripheral AS in this population. Our study indicated that ICVD patients with AS had a notably high prevalence of peripheral AS. We found that 9.9% of ICVD patients had RAS, which is similar to previous reports, and is significantly higher than the 0.5% seen in the general population^11,17^. However, the observed comorbidity rate of PAD at 25.3% not only surpasses the 2.9–4.1% prevalence rates in the general population but also exceeds the 12.2–18% prevalence rates reported in previous studies of ICVD patients^4,18,19^. This discrepancy can be attributed to our stringent inclusion and exclusion criteria, which excluded ICVD patients with a non-atherosclerotic etiology. Previous studies without distinguishing the etiology might have included non-atherosclerotic ICVD patients, potentially leading to an underestimation of this rate. Moreover, our study used lower limb arterial ultrasound instead of the commonly used ankle-brachial index (ABI), which is the ratio between ankle and arm systolic blood pressure when the patient is in a supine position^6^, to diagnose PAD. Ultrasound examination can directly assess the severity of luminal stenosis, significantly enhancing the sensitivity and specificity of PAD diagnosis compared to ABI^20,21^. Therefore, our approach potentially identifying more high-risk patients and thereby enabling their early interventions, so ultrasound examination is supposed to be used instead of ABI to screen PAD in ICVD patients with CAS.

As one of the systemic manifestation of AS affecting various vascular beds, PAD shares similar risk factors with CAS^22^. Previous studies have reported associations between PAD and ICAS and ECAS^9,23^. Our findings not only reveal a higher rate of CAS among patients with concomitant PAD but also suggest an association of the severity between PAD and both ICAS and ECAS. This underscores the importance of conducting a comprehensive assessment for the severity and extent of peripheral AS rather than merely determining its presence. Furthermore, while studies have reported that different locations of PAD (PADD/PADP) may be associated with the location of CAS, such as PAD was associated with ECAS rather than ICAS^24^. Our study found although there are differences in the occurrence rates of ICAS/ECAS among patients with PADP or PADD, regardless of whether the patients had PADD or PADP, the incidence rates of combined ECAS, ICAS, anterior circulation, and posterior circulation CAS were increased. This could be attributed to our relatively large sample size and a higher occurrence rate of CAS among the included patients. Analyzing PAD and CAS based on their location might offer a more precise assessment, and this correlation and its impact on prognosis requires further confirmation through larger clinical studies.

On the other hand, the relationship between RAS and CAS, although sharing many common risk factors and pathologies^25^, remains contentious^3,11–13,26^. Our results indicate an association between RAS and the degree of ECAS, but no significant correlation between RAS and ICAS was observed. One possible explanation could be the proximity in diameter between renal arteries and extracranial arteries, with both directly connected to large elastic vessels, exhibiting certain anatomical similarities^12^. Arteries of varying diameters differ in histological characteristics, antioxidant enzyme activity^27^, and levels of inflammatory markers^28^, among other aspects^12^. Although the potential mechanisms underlying these differences necessitate further validation through large-scale clinical experiments, our results suggested that it is necessary to strengthen RAS screening for ECAS patients.

Besides the traditional risk factors for AS, our study identified elevated fibrinogen and D-dimer as independent factors associated with the peripheral AS and CPAS in ICVD patients. Fibrinogen is related to the formation and development of AS, fibrinogen-fibrin promotes plaque growth by facilitating lipid deposition, attracting macrophages laden with low-density lipoprotein, and promoting smooth muscle cell proliferation^29^. Additionally, fibrinogen facilitates cholesterol transfer from platelets to monocytes/macrophages, promoting the production of foam cells, and unstable plaques contain more fibrinogen than stable plaques^30^. Furthermore, D-dimer, a soluble fibrin degradation product^31^, may also serve as a biomarker for AS because previous research has suggested that elevated D-dimer levels might be linked to the progression of PAD and an increased risk of IS and myocardial infarction^32,33^. These findings imply that fibrinogen and D-dimer might be significant associated with the development and progression of AS across different vascular beds, increasing the likelihood of multiterritorial AS, so it is necessary to pay attention to the detection and control of elevated fibrinogen to reduce the occurrence and progression of peripheral AS in ICVD patients. Nevertheless, further research is needed to fully understand the underlying pathophysiological mechanisms behind these associations.

This study has several limitations. Firstly, it is a single-center study with a relatively small sample size, which may restrict the generalizability of the results. Secondly, while renal artery ultrasound is the one of the current guideline-recommended diagnostic method for RAS^6^, it cannot directly assess AS plaques, potentially resulting in imprecise estimations of AS severity. And althrough CTA is currently the most accurate non-invasive vascular imaging method capable of simultaneously assessing cervicocephalic AS^34^ and ultrasound examination is more sensitive compared to the commonly used ABI, there are more sensitive or specific methods for imaging some specific arterial segments of cervicocephalic-peripheral arteries^34^. Employing a combination of imaging modalities maight enhance the precision of AS assessment and improve the accuracy of the results. Thirdly, our study lacks follow-up data. ﻿Longitudinal studies with extended follow-up periods are essential for assessing the progression of AS and elucidating the impact of PAD and RAS on the prognosis of ICVD patients. Meanwhile, research on antifibrinolytic therapy in patients with multiterritorial AS and high levels of fibrinogen should be conducted to clarify the influence of elevated fibrinogen and D-dimer on the prognosis of patients with multiterritorial AS. Additionally, our AS classification method remains relatively simplistic and may not include complete AS information. A more detailed quantitative assessment of the severity and extent of AS might help to more accurately analyze and judge the correlation of AS in different vascular beds.

## Conclusion

The occurrence rate of peripheral AS in ICVD patients with AS is approximately one-fourth, which is much higher than that in the general population. the degree of RAS was independently correlated with the degree of ECAS, while the degree of PAD was independently correlated with the degree of ICAS and ECAS. Besides traditional risk factors, elevated levels of fibrinogen and D-dimer are independent risk factors for peripheral AS and CPAS. Enhancing the assessment of peripheral AS and controlling elevated fibrinogen in ICVD patients might be helpful for preventing and delaying the progression of multiterritorial AS, thereby improving vascular risk stratification and prevention and treatment strategies.

### Supplementary Information

Supplementary Information.

## Data Availability

The datasets generated during and/or analysed during the current study are available from the corresponding author on reasonable request.

## References

[1] Katan M, Luft A (2018). Global burden of stroke. Semin. Neurol..

[2] Gutierrez J, Turan TN, Hoh BL, Chimowitz MI (2022). Intracranial atherosclerotic stenosis: Risk factors, diagnosis, and treatment. Lancet Neurol..

[3] Imori Y (2014). Co-existence of carotid artery disease, renal artery stenosis, and lower extremity peripheral arterial disease in patients with coronary artery disease. Am. J. Cardiol..

[4] Alberts MJ (2009). Three-year follow-up and event rates in the international REduction of Atherothrombosis for Continued Health Registry. Eur. Heart J..

[5] Olesen KKW (2017). Coronary artery disease and risk of adverse cardiac events and stroke. Eur. J. Clin. Investig..

[6] Aboyans V (2018). 2017 ESC Guidelines on the Diagnosis and Treatment of Peripheral Arterial Diseases, in collaboration with the European Society for Vascular Surgery (ESVS): Document covering atherosclerotic disease of extracranial carotid and vertebral, mesenteric, renal, upper and lower extremity arteriesEndorsed by: The European Stroke Organization (ESO)The Task Force for the Diagnosis and Treatment of Peripheral Arterial Diseases of the European Society of Cardiology (ESC) and of the European Society for Vascular Surgery (ESVS). Eur. Heart J..

[7] Campia U, Gerhard-Herman M, Piazza G, Goldhaber SZ (2019). Peripheral artery disease: Past, present, and future. Am. J. Med..

[8] Safian RD (2021). Renal artery stenosis. Prog. Cardiovasc. Dis..

[9] Barreto-Neto N (2016). Low ankle-brachial index is a simple physical exam sign predicting intracranial atherosclerotic stenosis in ischemic stroke patients. J. Stroke Cerebrovasc. Dis..

[10] Hong JB, Leonards CO, Endres M, Siegerink B, Liman TG (2016). Ankle-brachial index and recurrent stroke risk: Meta-analysis. Stroke.

[11] Kawarada O, Yokoi Y, Morioka N, Takemoto K (2007). Renal artery stenosis in cardio-and cerebrovascular disease: renal duplex ultrasonography as an initial screening examination. Circ. J..

[12] Wang K, Zhao JW, Jiang GM, Yun WW, Chen ZY (2013). Correlation of atherosclerotic renal artery stenosis with extracranial carotid and intracranial cerebral artery atherosclerosis in patients with ischemic stroke. Blood Press..

[13] Wu TC, Lee TH (2008). Low frequency of renal artery disease in young ischemic stroke patients. Acta Neurol. Taiwanica.

[14] Adams HP (1993). Classification of subtype of acute ischemic stroke. Definitions for use in a multicenter clinical trial. TOAST. Trial of Org 10172 in Acute Stroke Treatment. Stroke.

[15] Kong Q (2019). Patients with acute ischemic cerebrovascular disease with coronary artery stenosis have more diffused cervicocephalic atherosclerosis. J. Atheroscler. Thromb..

[16] Xu ZH, Sun XF, Zhang XD, Wang J, Ren JH, Wang X, Wang YH, Hou WH, Li JC (2021). Consensus on ultrasound diagnosis of renal artery stenosis. Chin. J. Med. Ultrasound (Electron. Ed.).

[17] Kuroda S (2000). Prevalence of renal artery stenosis in autopsy patients with stroke. Stroke.

[18] Rahman AS (2017). Ischaemic stroke and peripheral artery disease. JPMA J. Pak. Med. Assoc..

[19] Naito H (2016). Prevalences of peripheral arterial disease diagnosed by computed tomography angiography in patients with acute ischemic stroke. J. Stroke Cerebrovasc. Dis..

[20] AbuRahma AF (2020). Critical analysis and limitations of resting ankle-brachial index in the diagnosis of symptomatic peripheral arterial disease patients and the role of diabetes mellitus and chronic kidney disease. J. Vasc. Surg..

[21] Ugwu E, Anyanwu A, Olamoyegun M (2021). Ankle brachial index as a surrogate to vascular imaging in evaluation of peripheral artery disease in patients with type 2 diabetes. BMC Cardiovasc. Disord..

[22] Eraso LH (2014). Peripheral arterial disease, prevalence and cumulative risk factor profile analysis. Eur. J. Prev. Cardiol..

[23] Manzano JJ (2012). Associations of ankle-brachial index (ABI) with cerebral arterial disease and vascular events following ischemic stroke. Atherosclerosis.

[24] Shin YY (2019). Subclinical peripheral arterial disease in patients with acute ischemic stroke: A study with ultrasonography. J. Stroke Cerebrovasc. Dis..

[25] Safian RD, Textor SC (2001). Renal-artery stenosis. N. Engl. J. Med..

[26] Uzu T (2002). Prevalence and outcome of renal artery stenosis in atherosclerotic patients with renal dysfunction. Hypertens. Res. Off. J. Jpn. Soc. Hypertens..

[27] Bae HJ (2006). Correlation of coronary and cerebral atherosclerosis: Difference between extracranial and intracranial arteries. Cerebrovasc. Dis..

[28] Bang OY (2006). Intracranial atherosclerotic stroke: Specific focus on the metabolic syndrome and inflammation. Curr. Atheroscler. Rep..

[29] Hicks RC, Golledge J, Mir-Hasseine R, Powell JT (1996). Vasoactive effects of fibrinogen on saphenous vein. Nature.

[30] de Moerloose P, Boehlen F, Neerman-Arbez M (2010). Fibrinogen and the risk of thrombosis. Semin. Thromb. Hemost..

[31] Weitz JI, Fredenburgh JC, Eikelboom JW (2017). A test in context: D-dimer. J. Am. Coll. Cardiol..

[32] Soomro AY, Guerchicoff A, Nichols DJ, Suleman J, Dangas GD (2016). The current role and future prospects of D-dimer biomarker. Eur. Heart J. Cardiovasc. Pharmacother..

[33] Di Castelnuovo A (2014). Elevated levels of D-dimers increase the risk of ischaemic and haemorrhagic stroke. Findings from the EPICOR Study. Thromb. Haemost..

[34] Kong Q (2023). Atherosclerosis burden of brain- and heart-supplying arteries and the relationship with vascular risk in patients with ischemic stroke. J. Am. Heart Assoc..

