# Supplementary material for: Correlation and risk factors of peripheral and cervicocephalic arterial atherosclerosis in patients with ischemic cerebrovascular disease
Source: Sci Rep. 2024 May 23;14:11773. doi: 10.1038/s41598-024-62092-1 (PMC11116411; doi:10.1038/s41598-024-62092-1)
Supplement: Supplementary file 1 — Supplementary Information. [file 41598_2024_62092_MOESM1_ESM.pdf]

Supplementary Fig. S1. Baseline Characteristics of ICVD Patients

Characteristics	Total
Male (*n*, %)	281 (69.7)
Age (year, X ± S)	61.0±11.4
NIHSS on admission [M (Q25, Q75)]	1 (0-3)
History of HTN (*n*, %)	274 (68.0)
History of DM (*n*, %)	142 (35.2)
History of IS (*n*, %)	100 (24.8)
History of CAD (*n*, %)	60 (14.9)
Smoking (*n*, %)	173 (42.9)
Drinking (*n*, %)	148 (36.7)
BMI(kg/m^2, X± S)	25.7±3.4
Obesity (*n*, %)	236 (58.6)
SBP on admission (mmHg, X± S)	145.6±18.7
DBP on admission (mmHg, X± S)	89.6±14.9
HbA1C (%, X± S)	6.6±1.6
CK (IU/L, X± S)	97.8±200.8
Creatinine (umol/L, X± S)	63.2±15.0
Homocysteine (umol/L, X± S)	17.2±11.4
FBG (mmol/L, X± S)	6.3±2.4
Total cholesterol (mmol/L, X± S)	4.0±1.1
HDL-C (mmol/L, X± S)	1.1±0.3
LDL-C (mmol/L, X± S)	2.4±0.9
Apolipoprotein A1 (g/L, X± S)	1.2±0.2
Apolipoprotein B (g/L, X± S)	0.9±0.2
CRP (mg/L, X± S)	5.1±9.6
Fibrinogen (g/L, X± S)	3.2±0.9
D-dimer (ug/L, X± S)	1.7±4.5
Neutrophils (10^9/L, X± S)	4.2±2.1

Abbreviations: ICVD = ischemic cerebrovascular disease; NIHSS = National Institute of Health Stroke Scale; HTN = hypertension; DM = diabetes mellitus; IS = ischemic stroke; CAD = coronary artery disease; SBP = systolic blood pressure; DBP = diastolic blood pressure; BMI = Body Mass Index; HbA1C = glycosylated hemoglobin; CK = creatine kinase; FBG = fasting blood glucose; HDL-C = high density lipoprotein cholesterol; LDL-C = low density lipoprotein cholesterol; CRP = C reactive protein.

Supplementary Fig. S2. Correlation Analysis between Patients' Characteristics and the degree of ECAS and ICAS

Characteristics	The degree of ECAS		The degree of ICAS	
	r/Z	*p*	r/z	*p*
Sex	-1.628	0.104	-1.769	0.077**
Age	0.219	＜0.01*	0.203	＜0.01*
NHISS	0.023	0.647	0.080	0.107
History of HTN	-1.671	0.095**	-4.506	＜0.01*
History of DM	-0.822	0.411	-2.980	0.003*
History of IS	-2.207	0.027*	-3.875	＜0.01*
History of CAD	-1.427	0.154	-0.934	0.350
Smoking	-0.142	0.887	-0.534	0.593
Drinking	-0.710	0.478	-2.497	0.013*
SBP on admission	0.148	0.003*	0.119	0.017*
BMI	-0.071	0.157	-0.060	0.226
HbA1C	0.291	＜0.01*	0.135	0.007*
CK	-0.034	0.492	-0.036	0.467
Creatinine	0.026	0.606	0.006	0.908
Homocysteine	-0.001	0.989	0.039	0.442
FBG	0.249	＜0.01*	0.158	0.001*
Total cholesterol	0.076	0.126	-0.027	0.583
HDL-C	-0.077	0.125	-0.099	0.047*
LDL-C	0.100	0.044*	0.007	0.887
Apolipoprotein A1	-0.010	0.848	-0.099	0.048*
Apolipoprotein AB	0.167	0.001*	0.039	0.436
CRP	0.179	0.001*	0.130	0.015*
Fibrinogen	0.164	0.001*	0.186	＜0.01*
D-dimer	0.220	＜0.01*	0.133	0.008*
Neutrophils	0.063	0.208	0.063	0.209

* p value < 0.05 ** p value < 0.1

Abbreviations: ICVD = ischemic cerebrovascular disease; ECAS = extracranial atherosclerosis; ICAS = intracranial atherosclerosis; NIHSS = National Institute of Health Stroke Scale; HTN = hypertension; DM = diabetes mellitus; IS = ischemic stroke; CAD = coronary artery disease; SBP = systolic blood pressure; BMI = Body Mass Index; HbA1C = glycosylated hemoglobin; CK = creatine kinase; FBG = fasting blood glucose; HDL-C = high density lipoprotein cholesterol; LDL-C = low density lipoprotein cholesterol; CRP = C reactive protein.

Supplementary Fig. S3. Comparisons of clinical characteristics between ICVD patients with and without peripheral AS

Characteristics	Without peripheral AS(n=280)	With peripheral AS (n=123)	p
Male (*n*, %)	193(68.9)	88(71.5)	0.599
Age (year, X ± S)	59.1±11.6	65.2±9.7	＜0.01*
NIHSS[M (Q25, Q75)]	1(0-3)	2(1-3)	0.605
History of HTN (*n*, %)	176(62.9)	98(79.7)	0.001*
History of DM (*n*, %)	76(27.1)	66(53.7)	＜0.01*
History of IS (*n*, %)	61(21.8)	39(31.7)	0.034*
History of CAD (*n*, %)	35(12.5)	25(20.3)	0.042*
Smoking (*n*, %)	118(42.1)	55(44.7)	0.631
Drinking (*n*, %)	102(36.4)	46(37.4)	0.852
SBP on admission (mmHg, X± S)	143.8±19.3	149.9±16.5	0.002*
BMI(kg/m^2, X± S)	25.9±3.3	25.3±3.6	0.130
HbA1C (%, X± S)	6.3±1.4	7.3±1.9	＜0.01*
CK (IU/L, X± S)	106.0±236.9	79.5±65.7	0.224
Creatinine (umol/L, X± S)	62.9±14.4	63.8±16.0	0.599
Homocysteine (umol/L, X± S)	17.6±12.8	16.4±7.4	0.341
FBG (mmol/L, X± S)	5.9±2.0	7.1±3.0	＜0.01*
Total cholesterol (mmol/L, X± S)	4.0±1.1	4.1±1.1	0.088**
HDL-C (mmol/L, X± S)	1.1±0.3	1.0±0.3	0.272
LDL-C (mmol/L, X± S)	2.3±0.9	2.5±0.9	0.051**
Apolipoprotein A1 (g/L, X± S)	1.2±0.2	1.2±0.2	0.931
Apolipoprotein B (g/L, X± S)	0.2±0.2	0.9±0.2	0.002*
CRP (mg/L, X± S)	4.5±0.1	6.3±10.7	0.110
Fibrinogen (g/L, $\bar{X}$±s)	3.2±0.9	3.5±1.0	0.022*
D-dimer (ug/L, $\bar{X}$±s)	1.4±4.1	2.4±5.4	0.064**
Neutrophils (10^9/L, $\bar{X}$±s)	4.1±2.1	4.4±1.9	0.264

* p value < 0.05 ** p value < 0.1

Abbreviations: ICVD = ischemic cerebrovascular disease; AS = atherosclerosis; NIHSS = National Institute of Health Stroke Scale; HTN = hypertension; DM = diabetes mellitus; IS = ischemic stroke; CAD = coronary artery disease; SBP = systolic blood pressure; DBP = diastolic blood pressure; BMI = Body Mass Index; HbA1C = glycosylated hemoglobin; CK = creatine kinase; FBG = fasting blood glucose; HDL-C = high density lipoprotein cholesterol; LDL-C = low density lipoprotein cholesterol; CRP = C reactive protein.

Supplementary Fig. S4. Comparisons of clinical characteristics between ICVD patients with and without CPAS

Characteristics	Without CPAS(n=294)	CPAS(n=109)	*p*
Male (*n*, %)	202(68.7)	79(72.5)	0.464
Age (year, X ± S)	59.3±11.6	65.5±9.7	＜0.01*
NIHSS[M (Q25, Q75)]	1(0-3)	1(0.5-3)	0.744
History of HTN (*n*, %)	187(63.6)	87(79.8)	0.002*
History of DM (*n*, %)	80(27.2)	62(56.9)	＜0.01*
History of IS (*n*, %)	63(21.4)	37(33.9)	0.010*
History of CAD (*n*, %)	38(12.9)	22(20.2)	0.069**
Smoking (*n*, %)	125(42.5)	48(44.0)	0.784
Drinking (*n*, %)	108(36.7)	40(36.7)	0.994
SBP on admission (mmHg, X± S)	143.9±19.0	150±17.1	0.002*
BMI(kg/m^2, X± S)	25.9±3.3	25.3±3.6	0.103
HbA1C (%, X± S)	6.3±1.4	7.4±2.0	＜0.01*
CK (IU/L, X± S)	103.9±231.5	81.6±68.6	0.323
Creatinine (umol/L, X± S)	62.8±14.3	64.3±16.4	0.372
Homocysteine (umol/L, X± S)	17.6±12.6	16.2±7.1	0.266
FBG (mmol/L, X± S)	5.9±2.0	7.3±3.0	＜0.01*
Total cholesterol (mmol/L, X± S)	4.0±1.1	4.1±1.1	0.189
HDL-C (mmol/L, X± S)	1.1±0.3	1.0±0.3	0.209
LDL-C (mmol/L, X± S)	2.3±0.9	2.5±0.9	0.095**
Apolipoprotein A1 (g/L, X± S)	1.2±0.2	1.2±0.2	0.927
Apolipoprotein B (g/L, X± S)	0.8±0.2	0.9±0.2	0.008*
CRP (mg/L, X± S)	4.6±8.9	6.2±11.2	0.163
Fibrinogen (g/L, $\bar{X}$±s)	3.2±0.8	3.5±1.0	0.001*
D-dimer (ug/L, $\bar{X}$±s)	1.4±4.1	2.4±5.4	0.094**
Neutrophils (10^9/L, $\bar{X}$±s)	4.1±2.1	4.4±2.0	0.103

* p value < 0.05   ** p value < 0.1

Abbreviations: ICVD = ischemic cerebrovascular disease; CPAS = coexisting cervicocephalic-peripheral atherosclerosis; NIHSS = National Institute of Health Stroke Scale; HTN = hypertension; DM = diabetes mellitus; IS = ischemic stroke; CAD = coronary artery disease; SBP = systolic blood pressure; DBP = diastolic blood pressure; BMI = Body Mass Index; HbA1C = glycosylated hemoglobin; CK = creatine kinase; FBG = fasting blood glucose; HDL-C = high density lipoprotein cholesterol; LDL-C = low density lipoprotein cholesterol; CRP = C reactive protein.
